# Formulation of 5-fluorouracil loaded chitosan-based nanoparticles and evaluation of its cytotoxic effects against MCF-7 human breast cancer cells

**DOI:** 10.1371/journal.pone.0350929

**Published:** 2026-06-11

**Authors:** Idongesit Aniekan Ekpo, Airemwen Collins Ovenseri, Abdulrahman Abdullateef, Burak Durmaz

**Affiliations:** 1 Faculty of Pharmacy, Cyprus International University, Nicosia, Cyprus; 2 Department of Medical Biochemistry, Faculty of Medicine, Cyprus Health and Social Sciences University, Guzelyurt, Cyprus; 3 Department of Medical Biochemistry, Faculty of Medicine, Near East University, Nicosia, Cyprus; Laurentian University, CANADA

## Abstract

Breast cancer remains a major global health issue with a high mortality rate. In breast cancer treatment, chemotherapeutic drugs have a toxic effect on both cancerous and healthy cells which can lead to severe adverse effects and chemoresistance. Chitosan-based nanoparticles (NPs) have been reported to improve cellular drug delivery, thereby remediating the chemoresistance of anticancer drugs. The purpose of this research was to formulate 5-fluorouracil (5-FU) loaded chitosan nanoparticles. 5-FU nanoparticles were synthesized using ionic gelation technique which is based on the ionic interaction of positively charged chitosan and negatively charged tripolyphosphate that serves as a cross linker. The formulated nanoparticles were evaluated using UV spectrophotometer, Fourier-transform infrared spectroscopy, X-ray diffraction, Scanning Electron Microscopy. The cytotoxicity study was done using MTT assays. FTIR analysis revealed complete drug encapsulation of the nanoparticles via intermolecular interactions. The result of the XRD analysis showed that the drug was crystalline in the nanoparticle distribution. The SEM analysis confirmed the spherical morphology and structure. The synthesized nanoparticles had a mean particle size of 218.62 nm, zeta potential values of ≤+32.18 mV, Pdi values ranging from 0.14–0.29 and encapsulation efficiency results of ≤37.54%. The drug release mechanism was via Fickian diffusion. MTT analysis showed a time-dependent cytotoxic effect of 5-FU nanoparticles on breast cancer (MCF-7) cell lines after 72 h. This study confirmed that formulation of 5-FU into nanoparticles is a promising approach for sustained release and site-specific anticancer drug delivery which can potentially minimize the systemic side effects and improve treatment outcomes in cancer patients.

## Introduction

Breast cancer is among the most common causes of death for women between the age of 35–54years. Breast cancer, distinguished by distinct epidemiological patterns and considerable heterogeneity, continues to be a primary aetiology of cancer-related mortality among women [1,2]. Breast cancer (BC) terminologically also referred as breast carcinoma is a heterogenous disease in which cells of the breast grow out of control [3]. BC originates from the inner lining of the milk ducts or lobules [4].

Technological improvements, especially those using artificial intelligence and nanotechnology, have markedly enhanced the precision of breast cancer detection and diagnosis [5]. Significant advancements have been achieved in modern breast cancer treatment, leading to a continual decrease in breast cancer mortality rates and an improvement in the treatment outcomes [6].

Chitosan is one of the polymers mostly utilized in the manufacture of nanoparticles. Chitosan is connected by 1,4-glycosidic linkages, which can produce several active sites that interact with diverse groups [7]. Chitosan not only interacts with diverse hydrophilic molecules via hydrogen bonding or electrostatic interactions but also functions as a chelating agent, binding with metal ions to broaden its applications [8]. Chemotherapeutic agents are roughly classified into two primary categories depending on their modes of action, either cytotoxic or targeted medicines [9]. Cytotoxic medicines eradicate rapidly proliferating cancer cells by attacking elements of the mitotic and/or DNA replication processes [10].

The cytotoxic agent, 5-Fluorouracil (5-FU) is a pyrimidine antimetabolite that hinders DNA synthesis by inhibiting the enzyme thymidylate synthase responsible for the conversion of deoxyuridylic acid to thymidylic acid [11]. 5-FU is converted into cytotoxic metabolites that inhibit DNA and RNA synthesis, thereby preventing the proliferation of rapidly dividing cells and inducing programmed cell death (apoptosis) [12,13].

While 5-FU based chemotherapy represents a significant approach in cancer treatment, it is associated with drawbacks including systemic toxicity, limited effectiveness and selectivity, as well as the potential for resistance development [14]. It is a cytotoxic agent characterized by limited selectivity for cancerous cells [15]. It exposes healthy tissues to drug-induced toxicity when administered orally or via injection [16,17].

Majority of the cytotoxic drugs are non-selective and lack the ability to differentiate between cancerous and normal cells [18]. Nanoparticles have been previously reported to have the potential to overcome these challenges because of their small sizes and high surface area to volume ratio which can increase their cell permeability, targeting, half-life, bioavailability, and pharmacokinetic characteristics [19,20].

Due to selectivity, nanotechnology enables targeted drug delivery in the affected organs with low systemic toxicity [21]. Recently, the potential of nanotechnology to treat cancers has attracted a lot of attention due to its site-specific and targeted drug delivery [22]. Nanoparticles can be targeted to tumor cells via passive and active targeting technology due to their nanoscale size thereby reducing systemic side effects associated with chemotherapy and improving treatment outcomes for cancer patients. They are commonly used for the diagnosis, prevention and treatment of cancer [23]. In breast cancer treatment, chemotherapeutic drugs such as 5-FU tend to have a toxic effect on healthy cells and can lead to chemoresistance. To address the issue of inadequate selectivity, the systemic administration of these anti-cancer drugs is gradually being replaced by localized targeting and site-specific drug delivery, which have demonstrated promising outcomes in chemotherapy [24,25]. Previous studies have reported that there are different methods of treatment delivery for patients with breast cancer, and polymer-based nanoparticle therapy is one of the novel approaches used for standardized treatment [26,27]. Amalia et al., [28] reported that encapsulation of 5-FU into chitosan-based nanoparticles can significantly increase its cytotoxicity and targeted delivery compared to free 5-FU. Hence, the objective of this research was to formulate 5-FU loaded chitosan nanoparticles for increased selectivity, specificity and targeted drug delivery thereby reducing the severe adverse effects of the drug.

## Materials and methods

### Materials

Chitosan and 5-FU were both obtained from Sigma (Germany). Glacial acetic acid and sodium tripolyphosphate (TPP) acid were also procured from Merck (Germany). Deionized water was used in preparing all aqueous solutions. All other reagents were of analytical grade.

### Preparation of chitosan solution

Chitosan (1 g) was dissolved in 200 mL of acetic acid solution (1%v/v). The mixture was then stirred with a magnetic stirrer (RCT basic, Germany) at 600 rpm for 3 h at room temperature (25 ± 2°C) to obtain a proper dissolution and a clear, viscous solution which was sieved with a Whatman No. 1 filter paper, in order to remove the impurities. There was adjustment of the pH of the chitosan solution at about 5.0–5.5 using 1 M sodium hydroxide (NaOH) solution in achieving protonation of amine groups for adequate ionic cross-linking [29].

### Preparation of 5-fluorouracil solution

A solution of 5-FU was made by dispersing 2.0 g of the drug in 20 mL of distilled water. The solution was agitated at 400 rpm for 20 min at 25 °C, till a homogeneous concentrated 5-FU solution (100 mg/mL) was obtained [30].

### Preparation of Sodium Tripolyphosphate (TPP) crosslinker solution

A total 1 g of TPP was mixed with 40 mL of deionized water and agitated for 30 min. This then yielded a 25 mg/mL TPP solution which was preserved at room temperature and utilized for the nanoparticle synthesis [31]. TPP served as a cross-linker in the formulation [32].

### Synthesis of 5-FU loaded chitosan nanoparticles

The ionic gelation method was adopted in the preparation of 5-FU loaded chitosan nanoparticles by methods previously reported by Samy et al., [33]. 100 mg/mL of 5-FU solution was mixed with 50 mL of chitosan solution and stirred for 30 min. 25 mL of TPP solution was then added to the chitosan/5-FU mixture and stirred at 500 rpm using a magnetic stirrer for 1 h at 25 °C to allow the loading of the drug into the polymer matrix. The ratio of 5-FU: chitosan: TPP was 4:2:1 [34]. The nanoparticles preparation was centrifuged at 5,000 rpm for 30 min. The synthesized nanoparticles were lyophilized using a freeze dryer and collected. Three (3) batches of the 5-FU nanoparticles (5-FUNP1, 5-FUNP2 and 5-FUNP3) were formulated and batch 5-FUNP3 was adopted as the optimized formulation after some preliminary studies (Table 1).

**Table 1:** Formula of 5-FU loaded chitosan nanoparticles.

Formulation	5-FU (mg/mL)	Chitosan (mg/mL)	TPP (mg/mL)
5-FUNP1	25	12.5	6.25
5-FUNP2	50	25.0	12.5
5-FUNP3	100	50.0	25.0

### Characterization of the synthesized 5-FU loaded nanoparticles

#### Zeta Potential, Average Particle Diameter and Polydispersity Index (Pdi) analysis.

The nanoparticles (1 g) were mixed with 10 mL of distilled water and mixed continuously for 10 min. Malvern Zeta sizer Nano Sampler, was used to analyze the particle size distribution, zeta potential and Pdi of the synthesized nanoparticles. It was also utilized in the measurement of the surface charge marker of the nanoparticles at 25 °C [35].

#### Encapsulation efficiency (EE%).

The 5-FU nanoparticle (10 mg) was dissolved in 5 mL of 0.5% acetic acid and sonicated using a sonicator (Misonix, USA) for 25 min. The mixture was then centrifuged at 2500 rpm for 10 min. The quantity of free unencapsulated 5-FU in the supernatant layer was analyzed with the use of UV equipment (Shimadzu, Japan) at 264 nm. The entrapment efficiency (EE%) of the synthesized nanoparticles was determined using Equation 1 [36].


$$\begin{array}{c} \text{Entrapment efficiency }(\%)\;=\frac{\text{Total drug}-\text{Free drug}}{\text{Total drug}}\text{x 100 } \end{array}$$ (1)

#### Fourier-Transform Infrared (FTIR) spectroscopy analysis.

Pure sample of the drug and the optimized 5-FU loaded chitosan nanoparticles were analyzed for drug-excipient compatibility using FTIR spectrophotometer (Shimadzu, Japan) at 4000−400 cm^-1^ wavelength using the potassium bromide (KBr) method [37].

#### X-Ray Diffraction (XRD) analysis.

X-ray diffractometer (Rikagu, Japan) was used to analyze the crystallinity of the optimized 5-FU nanoparticles and the pure sample of 5-FU at a speed of 40kV, 40mA current intensity, 2θ diffraction angle in the range of 4–50°. The scanning parameters were program at 0.5 s scan step time [25].

#### Scanning Electron Microscopy (SEM) Analysis.

The shape of the synthesized 5-FU loaded chitosan nanoparticles and its nanoscale dimensions were analyzed using SEM (JEOL Co., China). The sample was gold coated, placed on a sample holder and examined at the appropriate magnifications

[38].

#### Differential scanning calorimetry (DSC).

A Differential scanning calorimeter (Mettler Toledo, USA) was used to thermally analyzed the pure sample of 5-FU and the synthesized 5-FU nanoparticles [39].

#### In vitro drug release study.

The in vitro release of 5-FU from the synthesized nanoparticles was analyzed using a dialysis cellulose membrane (Hi Media, India) by the methodology previously reported by Alhajj et al. [30] The percentage of drug released was computed.

#### In vitro drug release kinetics.

Zero and first order models as well as Higuchi, Hixson-Crowell cube root law and Korsemeyer-Peppas exponential models were used to compute the drug release kinetics [39,40].

### Cell Culture

MCF-7, an estrogen receptor-positive [ER (+)] breast cancer cell line, was obtained from American Type Culture Collection. Roswell Park Memorial Institute 1640 (RPMI-1640), containing 1% penicillin/streptomycin and 10% Fetal bovine serum (FBS), was used as a culture medium. Cells were plated in 25 and 75 cm^2^ flasks; in a 37 °C incubator supplying 5% partial CO_2_ pressure.

### Cytotoxicity Experiments

The technique previously reported by Durmaz et al., [41] was used. 3-(4,5-Dimethylthiazol-2-yl)-2,5-Diphenyltetrazolium Bromide (MTT) assay was utilized for the determination of cell viability and cytotoxicity investigation. MCF-7 cells (2x10^4^) were added into 96-well plates at a density of 3x10^3^ cells per well, respectively. After 24 h, different concentrations of Ch-5-FU compound (15.625, 31.25, 62.5, 125 and 250 µg/mL) were added to the cells. The wells without FU loaded nanoparticles were considered controls. A solution of MTT (2 mL) was added to each well at the end of 24, 48 and 72 h cycles. Optical density (OD) of each well was determined by absorbance at 570 nm wavelength using a microplate reader (Thermo-Scientific, Multiskan FC, Finland) for 4 h. The percentage of cytotoxicity value was determined by using the absorbance data, obtained from these experiments, and the following formula:


$$\begin{array}{c} \%\text{ cytotoxicity}=\text{100}-[\frac{\text{O}.\text{D of eperimental value}}{\text{O}.\text{D of control value}}]\times \text{100} \end{array}$$

The cytotoxic effect of the Ch-5-FU drug sample on breast cancer MCF-7 cell lines was determined in real time with the x Celligence RT-SP device.

### Statistical analysis

Results were presented as average ± standard deviation. GraphPad Prism Software Version 9.00 (GraphPad Software LLC, USA) was used for all analyses and P < 0.05 were considered statistically significant.

## Results

### Result of the UV-Vis analysis

The UV-Vis spectroscopy data is a basic step in confirming the formation of nanoparticles. The characteristic absorption peak for the drug loaded nanoparticles differs from the pure 5-FU peaks which indicates a successful interaction and encapsulation process confirming nanoparticles synthesis at a maximum wavelength of 264 nm. The alteration in the absorption profile indicates the formation of a new complex, thereby confirming the efficacy of the ionic gelation method. Samy et al., [32] also reported similar results.

### Result of the SEM analysis

**Fig 1:**
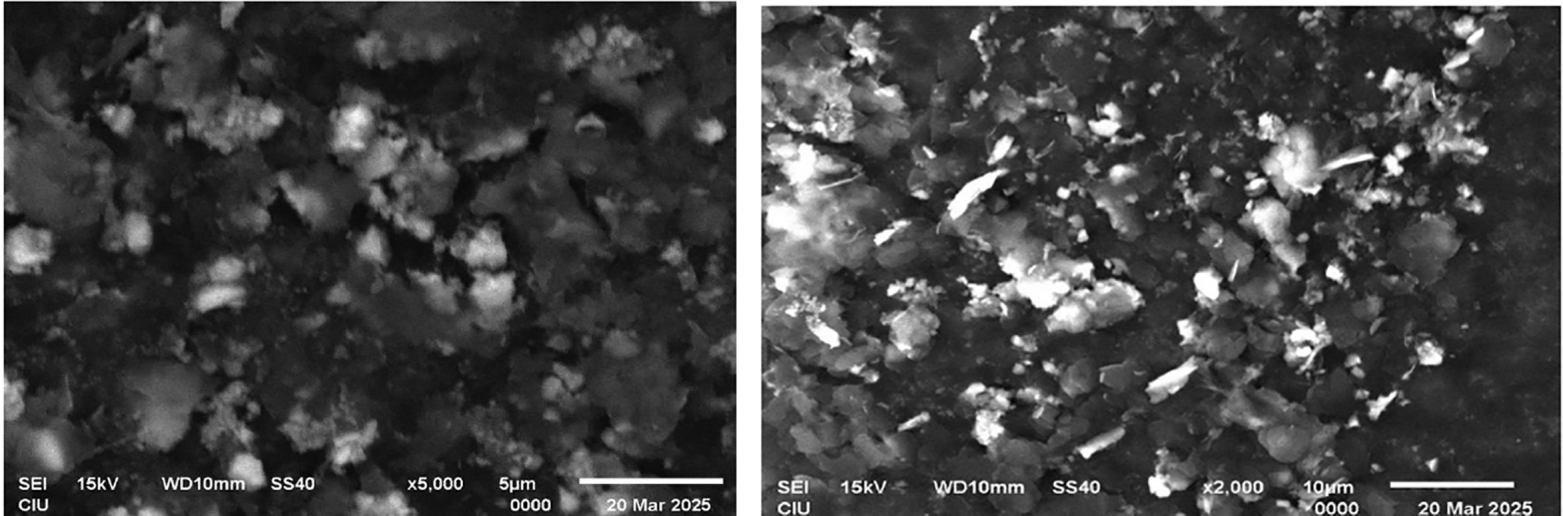
SEM images of (a) 5-FU pure sample (b) 5-FU loaded chitosan nanoparticles x10,000.

The SEM images of the pure sample and the optimized 5-FU nanoparticles are shown in Fig 1 and it revealed a spherical smooth and porous particles. Previous studies done by Samy et al., [32] reported similar findings. This may be due to rapid gelling ability of chitosan in presence of tripolyphosphate which is a polyanion forming both intramolecular and intermolecular cross linkages that result in the formation of stable nanoparticles.

### Results of Encapsulation efficiency (EE), Pdi, zeta potential and particle size analysis

The results of the encapsulation efficiency, zeta potential, Pdi and particle size analyses are shown in Table 2. The encapsulation efficiency results showed that the synthesized 5-FU chitosan nanoparticles had EE% values ranging from 28.42% to 37.54%.

**Table 2:** EE, Particle size, Pdi and Zeta potential Results of 5-FU Nanoparticles.

Formulation	EE ± SD (%)	Particle size±SD (nm)	Pdi ± SD	Zeta potential±SD (mV)
5-FUNP1	28.42 ± 0.01	108.41 ± 0.01	0.14 ± 0.02	+40.27 ± 0.01
5-FUNP2	31.68 ± 0.02	95.72 ± 0.01	0.19 ± 0.01	+42.12 ± 0.01
5-FUNP3	37.54 ± 0.01	97.74 ± 0.01	0.29 ± 0.01	+32.18 ± 0.01

The size analysis results showed that all the synthesized 5-FU nanoparticles were in the range of nanoscale with the particle sizes ranging from 148.41 ± 0.01–287.74 ± 0.01 nm. The result of the Pdi analysis showed that all the synthesized nanoparticles had Pdi values ranging from 0.14–0.29. Th formulations had a Pdi value of < 0.3 indicating a monodisperse and narrow size distribution. [31] The Zeta potential values of the synthesized 5-FU nanoparticles ranged from +24.27 ± 0.01 to +32.18 ± 0.01 mV indicating homogenous particle distribution.

### Result of the DSC analysis

**Fig 2:**
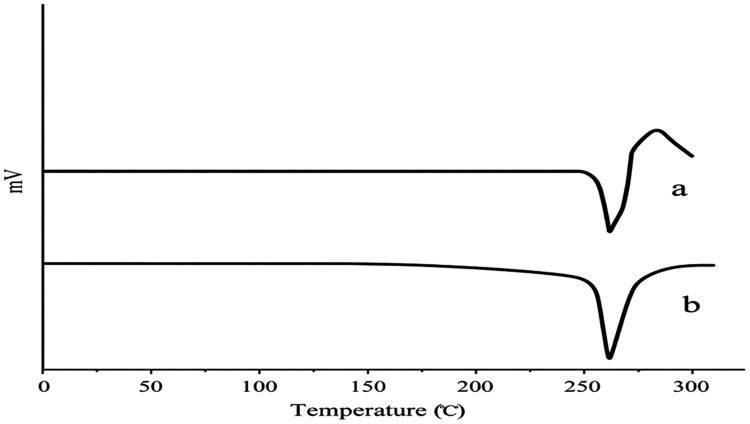
DSC thermogram of (a) 5-FU pure sample and (b) 5-FU loaded nanoparticles.

The DSC thermograms of the synthesized nanoparticles and that of the pure 5-FU are shown in Fig 2. DSC technique gives vital information on the physicochemical properties of the drug incorporated into nanoparticles [42]. The DSC thermogram of 5-FU showed endothermic peaks at 285.79 °C and 295 °C which corresponds to the melting point of the drug while the DSC thermograms of the optimized nanoparticles showed a disappearance of the distinct 5-FU endothermic peak at 295 °C.

### Result of the XRD analysis

**Fig 3:**
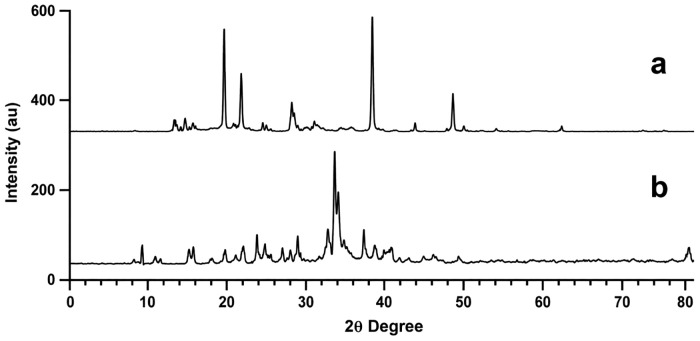
XRD spectra of (a) pure 5-FU sample and (b) 5-FU loaded chitosan nanoparticles.

The XRD spectra of the pure 5-FU and the synthesized5-FU nanoparticles are shown in Fig 3. The XRD spectrum of pure 5-FU revealed strong several diffraction peaks at 2θ = 16.1, 19.8, 22.1, 28.4, 32.7, 40.1 and 46.5°, which correspond to 1000, 220, 500, 470, 490, 180 and 205 lattice planes respectively which indicates the crystallinity of the drug [43]. The slight disappearance of some of the strong peaks at 20.0°, 22.1° and 45.2° in the spectra of the drug loaded chitosan nanoparticles indicates the encapsulation of 5-FU within the chitosan polymer.

### Result of the FTIR analysis

**Fig 4:**
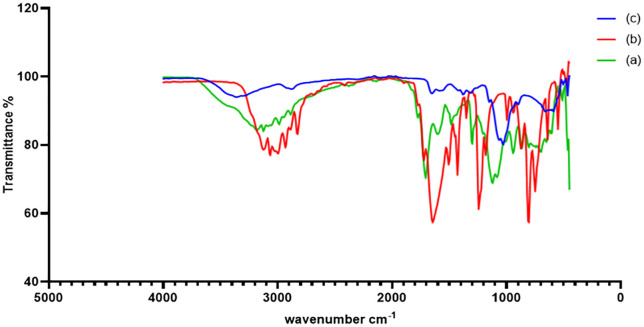
FTIR spectra of (a) 5-FU, (b) 5-FU loaded nanoparticles and (c) chitosan.

The FTIR spectra of chitosan, pure 5-FU and the optimized 5-FU loaded chitosan nanoparticles are displayed in Fig 4. The FTIR spectrum of chitosan revealed characteristic peaks at 652.4, 857.7, 1124.8, 1726,4, 2954.8, and 3487.5 cm^-1^ due to the resonance of the C–O–C, O–H, N–H, C–O, -C-N stretching indicating amide I and polysaccharide backbone of chitosan.

The FTIR spectrum of 5-FU revealed distinct peaks at 658.5, 710.8, 852.1, 948,2, 1345.9, 1542.2, 1782.9 and 3128.5 cm^-1^ due to the vibration of the C = O, C = C, -C-N, -C-F functional groups indicating imide vibrations (amide II and III), carbonyl groups, aromatic and pyrimidine ring vibrations [44].

The FTIR spectrum of 5-FU nanoparticles was similar to that of the pure 5-FU and it revealed distinct peaks for both the drug and the polymers used in the formulation of the nanoparticles (chitosan and tripolyphosphate). There was a shift in the peaks of the spectrum to 712.9. 1008.2, 1150.4, 1,354.2, 1806.1 and 3216.4 cm^-1^.

### Results of the drug release study

**Fig 5:**
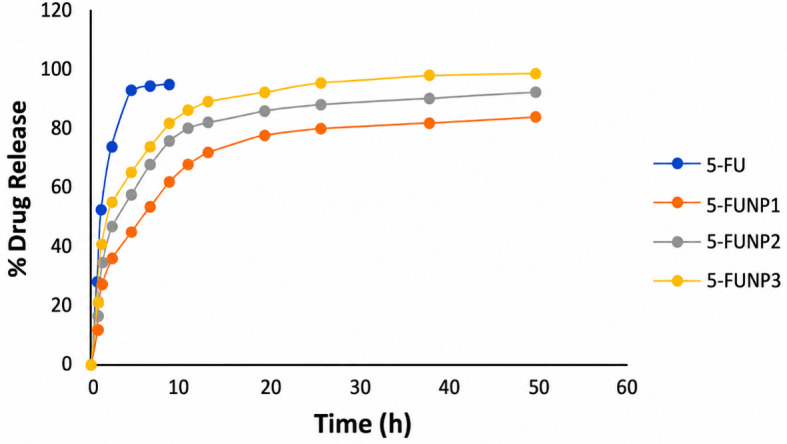
Drug release profile of free 5-FU dispersion and the three batches of drug loaded nanoparticles.

The in vitro release profile of 5-FU from the three (3) batches of drug loaded nanoparticles formulated in the study and the 5-FU dispersion are shown in Fig 5. The 5-FU loaded nanoparticles formulations displayed an extended drug release profile where the percentage drug released after 48 h was 84.28%, 92.87% and 99.12% for 5-FUNP1, 5-FUNP2, and5-FUNP3, respectively while 5-FU dispersion showed an immediate drug release of 93.45% after 4 h.

### Results of the drug release kinetics

The release kinetics of the formulated 5-FU loaded chitosan nanoparticles fitted most to Higuchi release model (Table 3). This shows that all batches of the formulations simulated diffusion-based release kinetics which is similar to the report of a previous study done by Alhajj et al. [30]

**Table 3:** Results of the in vitro 5-FU release kinetics.

Models	Zero	First	Higuchi	Hixon Crowell	Baker Lonsdale		Korsmeyer and Peppas
**Formulations**	**r** ^**2**^	**r** ^**2**^	**r** ^**2**^	**r** ^**2**^	**r** ^**2**^	**r** ^**2**^	**n**
5-FUNP1	0.827	0.921	0.974	0.872	0.884	0.371	0.40
5-FUNP2	0.849	0.948	0.982	0.894	0.881	0.387	0.41
5-FUNP3	0.858	0.954	0.989	0.897	0.897	0.379	0.44

### Results of the cytotoxicity assay

**Fig 6:**
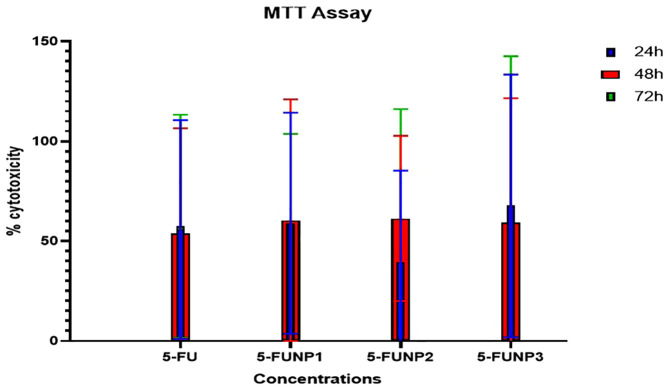
Cytotoxic effects of the 5-FU nanoparticles on breast cancer (MCF-7) cell lines after 24, 48, and 72 h. Values are mean ±SD n = 3. Significantly different from control (P < 0.05).

MTT colorimetric assay is commonly used in cell biology to determine cell viability, cell metabolic activity and effect of cytotoxic drugs on cells. The results of the dose-related % cytotoxicity and IC_50_ values on human breast adenocarcinoma cell lines, for the 24, 48, and 72 h are shown in Fig 6.

The 24 h MTT assay showed moderate cytotoxicity of drug loaded chitosan nanoparticles on breast cancer cells. The relatively high IC_50_ value of 95 µg/mL suggests it is not a highly potent toxic agent. The results revealed a dose-dependent increase in cell death. The 48-h MTT assay showed a significantly higher cytotoxicity effect of drug loaded chitosan nanoparticles on breast cancer cells compared to the effect after 24 h. The IC_50_ value reduced from 95.28 µg/mL to 47.35 µg/mL, indicating that the synthesized 5-FU nanoparticles demonstrated increased potency with longer exposure hence a lower concentration was required to inhibit 50% of the MCF-7 cells. The 72 h MTT assay demonstrated a better time-dependent cytotoxic effect. The IC_50_ further decreased to 24.50 µg/mL indicating that 5-FU loaded nanoparticles potency increased significantly with longer exposure (P < 0.05).

## Discussion

**Fig 7:**
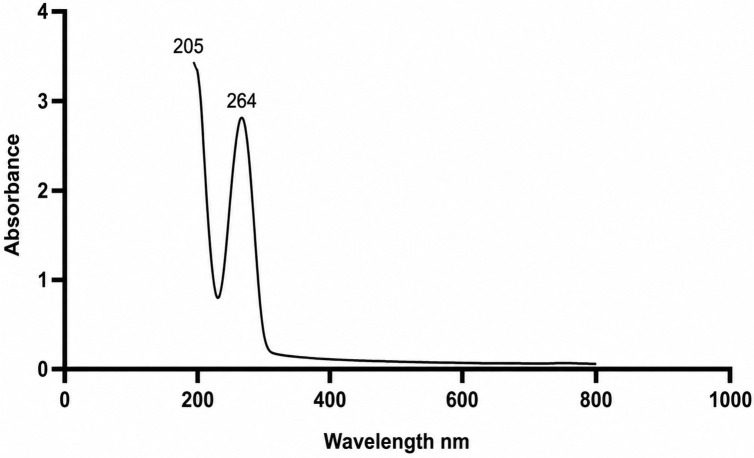
UV-Visible spectrophotometry of 5-FU loaded nanoparticles.

The synthesis of 5-FU nanoparticles in this study was due to the principle of ionic interaction of positively charged chitosan and negatively charged tripolyphosphate. The use of TPP as chitosan crosslinker is a validated method that has been previously reported in the formulation of spherical, smooth and stable nanoparticles where TPP is bonded to chitosan amino groups electrostatically to produce ionically crosslinked nanoparticles [45]. According to a study done by Samy et al., [37] the visible change in the appearance of the chitosan solution following the addition of TPP is a strong indication that the physical state of chitosan has been transformed molecularly from a clear solution to a colloidal dispersion of nanoparticles. The results of the UV spectroscopy showed a successful incorporation of 5-FU into the polymer confirming nanoparticles synthesis at a maximum wavelength of 264 nm (Fig 7).

The result of the encapsulation efficiency was ≤ 37.54%. EE % significantly increased as the concentration of chitosan was increased in the formulations (p < 0.05). The results showed that the concentration of chitosan as well as chitosan:TPP ratio had a significant effect in the encapsulation efficiency of the drug. This result agrees with previous studies done by Nagarwal et al., [46,47] as they reported that an increase in the amount of chitosan from 1–3 mg/mL, resulted in a corresponding increase in the entrapment efficiency of the drug. Samy et al., [32] also reported an increase in encapsulation efficiency with the increase of chitosan concentration from 1 to 4 mg/mL. This significant increase in the encapsulation efficiency with increasing chitosan concentration may be due to electrostatic attraction and adsorption between the drug and chitosan molecules [40]. Formulation 5-FN3 showed the highest 5-FU loading ability and encapsulation efficiency.

The mean particle size of the synthesized nanoparticles was 218.62 nm. Nanoparticles with sizes between 100–300 nm have been reported to have the enhanced permeability and retention (EPR) effect, which allows their accumulation in tumor tissues due to permeable vasculature and impaired lymphatic drainage of solid tumors [43]. The size achieved in this study was suitable for cellular uptake via endocytosis. Also, an increase in the chitosan concentration resulted in an increase in the particle size of the nanoparticles. The increase of particle size due to an increase in chitosan concentration may be attributed to higher viscosity during ionic gelation and reduced intermolecular distance between chitosan molecules at higher concentrations, resulting in the synthesis of larger particles [37].

The results of the zeta potential and pdi were ≤+32.18 mV and ≤0.14 respectively which indicate good charge conduction. This is an indication of the stability of the synthesized nanoparticle dispersion. Baetke et al., [48,49] reported that a sample with a high zeta potential with a threshold (> ±25 mV) generates high electrostatic repulsion within particles, which prevents aggregation and ensures colloidal stability. Pdi is an important term that explains the size distribution of the particles. Values can range from 0 to 1, with values <0.3 indicating monodisperse particles and values >0.3 indicating polydisperse particle size distributions [25].

The stability of nanoparticles distributed in aqueous vehicles has been found to be influenced by electrostatic and stearic charges, and a high zeta potential value denotes good stability in the colloidal dispersion. Hence, the synthesized nanoparticles were comparatively stable and monodispersed (Table 2) [43].

DSC determines variation in enthalpy due to changes in the physical and chemical effects of a drug as a function of temperature. The result of the DSC thermogram of the optimized nanoparticles showed a slight alteration of the distinct 5-FU endothermic peak at 295 °C. Previous studies reported a similar result and this may be as a result of the drug loading within the nanoparticles at a molecular level (Fig 2) [36].

The XRD analysis showed that the pure 5-FU is a crystalline compound with visible, sharp peaks in its XRD spectrum while there was a reduction in the intensity of the peaks in the XRD spectrum of 5-FU nanoparticles which indicates the encapsulation of 5-FU within the chitosan polymer (Fig 3). This resulted in the change of 5-FU from a crystalline to an amorphous state [50]. Previous studies have reported that the amorphous form of a drug generally has better solubility and dissolution rates and by extension better bioavailability and therapeutic efficacy. Previous studies also reported similar findings for other drugs incorporated into chitosan nanoparticles [31].

The FTIR results showed a slight shift in the position of the unique C-F bond stretching which showed 5-FU characteristic peak between 1285−1298 cm^-1^ indicating successful drug encapsulation and nanoparticles synthesis. The results revealed that there was a conjugation between the 5-FU and the chitosan. It is also showed that both the drug and ingredients used in the formulation of the nanoparticles were compatible (Fig 4). A similar result was also reported in a study done by Ozturk et al., [42].

The release profiles of the drug loaded nanoparticles exhibited a biphasic drug release pattern, with an initial immediate drug release within 1 h and a subsequent sustained, delayed drug release for 48 h (Fig 5). This sustained drug release of 5-FU was previously reported from chitosan-based polymers and may be due to the strong hydrogen bond between the 5-FU molecules, chitosan and tripolyphosphate which retarded the diffusion of the drug from the polymer matrix into the dissolution medium [45]. The percentage drug released significantly increased with an increase in the amount of chitosan (*P < 0.05*). Previous studies done by Samy et al., [32] also reported a similar finding that an increase in the molecular weight of chitosan resulted in a more sustained release drug release from chitosan-based nanoparticle formulation.

The drug release from the nanoparticle formulations simulated the Higuchi release model. The results of the release exponent (n) obtained in this study were ≤ 0.43 meaning that the mechanism of drug release was via Fickian diffusion (Table 3).

The cytotoxicity results revealed a decrease of the IC_50_ values from 95.28 µg/mL at 24 h to 47.35 µg/mL at 48 h and 24.50 µg/mL at 72 h which implies that 5-FU loaded chitosan nanoparticles demonstrated a potent dose-related and time-dependent cytotoxic effect against breast cancer cell lines compared to the control group that displayed no significant cytotoxic effect against the MCF-7 cell lines used in the study (Fig 6). The consistent non-cytotoxic effect of the control group over time validated the cytotoxicity assay, which affirms that apoptosis seen in the treated groups were genuinely due to the effect of the drug loaded nanoparticles (Fig 6). Amalia et al., [33] also reported a significant increase in the cytotoxic effect of 5-FU after 24 h and 48 h treatment with an IC_50_ of 3.1 ± 0.9 µM for all doses. 5-FU has been reported to induce apoptosis by inhibition of the enzyme, thymidylate synthase, interference with DNA/RNA synthesis thereby causing programmed cell death [40]. Previous studies have also reported that a combination of 5-FU with other anticancer agents such as paclitaxel and doxorubicin can produce a synergistic effect, resulting in decreased cell viability and increased apoptosis [44].

## Conclusion

The synthesized 5-FU loaded chitosan nanoparticles were stable, nanoscale in size, spherical, and demonstrated a time-dependent cytotoxicity against breast cancer cells. The findings of this research showed that 5-FU nano-formulation may address some the shortcomings of the conventional systemic 5-FU therapy such as non-selectivity, rapid systemic elimination, and the emergence of chemoresistance. This study demonstrated that formulation of 5-FU into nanoparticles is a viable approach for sustained release and cancer chemotherapy which can minimize the systemic side effects and improve treatment outcomes in cancer patients.

### Limitations

A limitation of this study is the absence of drug-free nanoparticles in DSC and XRD analyses, which would have provided clearer differentiation between polymer and drug-related thermal and crystalline transitions.
